# Association of the systemic immune inflammation index with failure after core decompression for osteonecrosis of the femoral head: a prospective time-to-event analysis

**DOI:** 10.1080/07853890.2025.2566867

**Published:** 2025-10-06

**Authors:** Chengsi Li, Haichuan Guo, Ziyu Han, Tianyu Wang, Dongwei Wu, Zhenbang Yang, Xinqun Cheng, Yingze Zhang, Yanbin Zhu

**Affiliations:** ^a^ Department of Orthopaedic Surgery, Hebei Medical University Third Hospital, Shijiazhuang, Hebei, P.R. China; ^b^ School of Basic Medical Sciences, the Hebei Medical University, Shijiazhuang, Hebei, P.R. China; ^c^ Orthopedic Research Institute of Hebei Province, Key Laboratory of Biomechanics of Hebei Province, Shijiazhuang, Hebei, P.R. China

**Keywords:** Core decompression, systemic immune inflammation index, osteonecrosis of the femoral head, prospective study, cumulative rate

## Abstract

**Objective:**

This study aimed to assess the association between preoperative systemic immune inflammation index (SII) and failure after core decompression (CD).

**Methods:**

We conducted a prospective study of patients admitted to a tertiary referral hospital with osteonecrosis of the femoral head who underwent CD between October 1, 2014 and April 30, 2019, and provided a minimum 3-year follow-up assessment. Restricted cubic splines assessed the dose–effect relationship between SII and failure. Propensity score matching (PSM) balanced potential preoperative confounders. Kaplan–Meier analyses estimated cumulative incidence as a function of time for the failure between low SII and high SII groups. Multivariable Cox proportional hazards models evaluated the independent association of high SII with failure after adjustment for perioperative factors. Prespecified subgroup analyses explored heterogeneity.

**Results:**

We found a positive relationship between the preoperative SII and failure after CD. Among 963 CD procedures performed in 676 patients, failure was observed in 97 cases in a median period of 5.4 years. The cumulative incidence of failure was 10.6% (95% CI, 8.7%, 12.8%) at 5 years. After PSM, failure rates at 5 years were identified as significant differences between the high SII and low SII groups (Log-rank p = 0.019), and that high SII was independently associated with a 1.90-fold (95% CI, 1.14, 3.18; p = 0.014) increased risk of failure. Significant heterogeneity was observed by gender and aetiology (P for interaction < 0.05).

**Conclusions:**

Preoperative high SII is a significant risk factor for failure after CD, which could be considered when evaluating surgical indications and providing preoperative counseling to patients.

## Introduction

Nontraumatic osteonecrosis of the femoral head (ONFH) is a common orthopedic disease characterized by chronic inflammation, primarily affecting those aged 30–50 years [1,2]. Among younger patients with early to mid-stage disease, core decompression (CD) has been recommended as the standard hip-preserving procedure [3–5]. However, its highly variable failure rates (16.0% to 34.0%) and timing (0.2 to 19 years) persistently plague surgeons [6,7]. Early failure cases are inevitably conversion to arthroplasty, which is associated with prosthesis infection, lower patient satisfaction, and revision surgery [8]. Nevertheless, failure related predictors remain sparse, and the existing radiological assessments have limited predictive capacity [9,10]. Therefore, it is imperative to find additional reliable prognostic assessments to reduce or even prevent early failures.

Assessing the patient’s systemic inflammatory status through peripheral blood parameters offers a promising direction. The widely recognized explanation is that chronic systemic inflammation, triggered by damage-associated molecular patterns, creates an ongoing environment of immune activation that impedes neovascularization and osteogenesis [1]. Several studies attempted to find the association between inflammatory variables and ONFH [11–13], but were limited by the ability to reflect only a portion of intrinsic mechanisms in a single variable, and only weak statistical correlations could be found. The systemic immune inflammation index (SII) is a novel composite index integrating two independent leukocyte subpopulations and platelets, reflecting the interaction of thrombocytosis, inflammation, and immunity, which is easily and readily available from routine laboratory tests [14]. In fact, the SII has been widely confirmed to be significantly associated with a poor prognosis in various medical and surgical specialties (e.g. orthopedic trauma, cancer, cardiovascular disease) [14–18]. In terms of orthopedic surgeries, SII was shown to be significantly associated with poor all-cause mortality in older adults after hip fracture surgeries [16]. However, to our knowledge, no studies have investigated the association between SII and hip preservation failure after CD.

Therefore, our objective was to conduct a prospective cohort study to explore and quantify the true association between preoperative SII and failure after CD, which could provide a new direction for earlier screening and surgical decision-making for ONFH.

## Methods

### Data source

This study used data derived from the secondary analysis of the Surgical Site Infection in Orthopedic Surgery database, which has been maintained prospectively since October 1, 2014, in a major teaching hospital and tertiary referral center in central China. The database, updated yearly by more than 230 trained medical staff (comprising trained residents and medical students with standard training), includes extracting administrative and clinical data from electronic medical records, retrieving imaging examination data from the Picture Archiving and Communication System, and obtaining laboratory results from the Laboratory Information System. Follow-up evaluations were performed at 2 weeks, 1 month, 3, 6, and 12 months postoperatively, by telephone interviews or outpatient visits. The database, widely used by orthopedic researchers [19–24], offers greater accuracy than administrative databases because it is manually curated and updated.

### Study design and populations

This prospective cohort study included patients who underwent CD at a tertiary referral orthopedic center from October 2014 to April 2019. These patients were 18 years or older, with Association Research Circulation Osseous (ARCO) stages I - IIIA ONFH. Exclusion criteria included a history of fracture in the proximal femur or tumors, history of any surgery on the affected hip, inflammatory arthritis (including suppurative arthritis and gouty arthritis), recent steroid treatment within the last 6 months [25], pregnancy, underwent other hip-preserving procedures, and incomplete data. Before surgery, surgeons confirmed MRI-based ARCO stage and lesion morphology (modified Kerboul angle [mKA] and Japanese Investigation Committee [JIC] type) and offered multiple drilling CD to symptomatic pre-collapse hips (ARCO I–II). CD alone was preferred for small-to-medium, non-lateral lesions, whereas larger and/or lateral lesions (particularly JIC C2) were treated with CD in conjunction with bone grafting. Selected early IIIA hips with minimal, non-flattening collapse were considered for CD only in conjunction with bone grafting [4,10]. Postoperatively, all patients were instructed to perform non-weight bearing functional exercises in the CD limb for at least 6 months. Patients who underwent simultaneous contralateral total hip arthroplasty (THA) were required to carry partial weight on the THA limb in 1 week and full weight in 2 weeks. The sample size should be at least 10 times the number of variables; with 30 variables in this study, the minimum required sample size was 300 [26]. The study adhered to the Declaration of Helsinki and the Strengthening the Reporting of Observational Studies in Epidemiology guidelines. Informed consent was obtained from all participants or their legal guardians during hospitalization.

### General information

All data for this study were obtained from medical records and follow-up visits. Preoperative baseline characteristics comprised demographic characteristics, physiological status, and disease severity, including age, gender, body mass index (BMI), living place, current smoking, aetiology, age-adjusted Charlson’s comorbidity index (aCCI), ARCO stage; American Society of Anesthesiologists (ASA) class and preoperative comorbidities, including hyperlipidemia, hypertension, diabetes, cerebrovascular disease, heart disease, lung disease, liver disease, kidney disease, tumor, peripheral vascular disease, and connective tissue disease. BMI was calculated based on the self-reported height and weight of the patient or his family. Perioperative data included total hospital stay, surgical side, thrombosis, anticoagulation management, (with or without) simultaneous contralateral THA procedure, year of surgery, surgeon experience, anesthesia method, and type of graft.

### Assessment of SII

Parameters were extracted from the routine blood test and biochemical tests performed before surgery, whichever came first. These laboratory variables were assessed using manufacturer-recommended methods. Biochemical tests were performed using a Beckman Colter AU5800 chemistry analyzer, while blood cell count tests were performed on a UniCel DXI 800 (Beckman Colter). SII was calculated from platelet counts (reference range: 100–300 × 10^9/L), neutrophil counts (reference range: 4–10 × 10^9/L), and lymphocyte counts (reference range: 1.1–3.2 × 10^9/L), using the following formula: SII = platelet × neutrophil/lymphocyte counts, as defined previously [14]. The SII was expressed as × 10^9/L.

### Follow-up and outcome

We conducted a prospective evaluation for these operated patients for CD between October 1, 2014 and April 30, 2019. We followed patients from the date of the procedure until the first of the following events to occur: failure, loss of follow-up, death, or until April 30, 2022, providing a minimum potential follow-up period of 3 years. The outcome of this study was the time to failure and was defined as conversion of CD to subsequent surgery at the last follow-up of the patients. For each patient, we identified the presence or absence of subsequent surgery using operative reports and patient radiographs available in the electronic medical record system of our institution, or medical records obtained from those other hospitals.

### Statistical analysis

To assess the distribution of continuous variables, we applied both the Shapiro–Wilk and Kolmogorov–Smirnov tests, with the latter performed after z-score normalization. Visual inspections were also conducted using histograms with kernel density curves and Q–Q plots (Supplementary Figure 4). All continuous variables were conservatively summarized as medians [Q1–Q3] and compared using the Mann–Whitney U test due to significant results in normality tests. Categorical variables were expressed as numbers (%) and analyzed with the χ^2^-test. The optimal age cut-off point was determined at 47 through the function surv_cutpoint. Missing BMI data (4.8%) were imputed using multiple imputations. Age and BMI were then converted into categorical variables for subsequent Cox regression analyzes.

To explore the dose-effect relationship between SII and failure after CD, we utilized a restricted cubic spline (RCS) model with four knots. To ensure the accuracy of the relationship between SII and failure risk, we adjusted for all covariates with a P-value less than 0.1 in univariate analysis among baseline characteristics (age, place of residence, aCCI, hyperlipidemia, ARCO stage, anesthesia method, and ASA class). The adjusted RCS model revealed that the risk of failure after CD began to exhibit a significant upward trend when the SII value reached 588.39. Then we designated SII = 588.39 as the reference threshold to categorize patients into two groups: a low SII group (< 588.39) and a high SII group (≥ 588.39). To minimize confounding from preoperative baseline characteristics, propensity score matching (PSM) was performed using 1:1 nearest neighbor matching algorithm with a 0.01 caliper width. Standardized mean differences < 0.1 indicated adequate group balance [27]. Before and after PSM, Kaplan-Meier analyses were performed to estimate cumulative incidence as a function of time for the failure of CD between low SII and high SII groups, with Log-rank and Mantel-Cox analyzes. Multivariate Cox proportional hazards model was fitted to assess the significance and magnitude of the association between high SII and failure, where perioperative factors were incorporated for further adjustment. Proportional hazards assumption was evaluated graphically, using log-log plots and Schoenfeld residual plots (Supplementary Figures 1–2). Potential population heterogeneity was explored by testing the interaction between high SII and predefined subgroups: age (years, < 47 vs. ≥ 47), gender (male vs. female), BMI (Kg/m2, < 28 vs. ≥ 28.0), living place (rural vs. urban), aetiology (steroid vs. alcohol vs. idiopathic) aCCI (0 vs. 1–2 vs. ≥ 3), ASA class (I–II vs. ≥ III), and ARCO stage (I vs. II vs. IIIA)[28].

To ensure result robustness, we applied inverse probability treatment weighting (IPTW) and conducted sensitivity analyses on post-PSM data by: adjusting HRs that accounted for pair matching after PSM; adjusting HRs that accounted for clustering among bilateral procedures; incorporating lung disease in the model to adjust the imbalance after PSM; stratifying by surgeon experience; excluding patients with simultaneous contralateral THA; excluding patients with missing BMI; excluding patients with CCI greater than or equal to 3; performing Landmark analysis (excluding early failures within 12 months post-operation with survival time recalibration). Competing risk analysis was unnecessary due to the low mortality rate in our sample.

We re-reviewed preoperative MRIs in the post-PSM cohort to obtain the mKA, JIC type, and bone marrow edema (BME) status, excluding hips with missing data along with their matched pairs. Three post-hoc analyses were then performed: to ensure morphological comparability in the pre-collapse stage, efficacy analyses were restricted to ARCO I–II hips (*n* = 561), (1) subgroup analyses were conducted by mKA grade and JIC type; and (2) location-stratified Cox models were constructed for JIC types C1 and C2 separately, with mKA adjusted continuously using restricted cubic splines. Both models adjusted for perioperative covariates. To eliminate potential bias from subclinical collapse, we further (3) excluded hips with preoperative BME and reassessed the main outcome in the BME-negative subgroup.

### Power analysis

To evaluate the statistical power of our primary analysis, we performed post hoc power calculations using the powerSurvEpi package, under a Cox proportional hazards model. The assumed effect size was derived from the observed HR between high and low preoperative SII groups in relation to the risk of conversion to subsequent surgery. In the unmatched full cohort (*n* = 963), with a HR of 1.84, the statistical power to detect this association at a two-sided alpha level of 0.05 was 86.0%. In the PSM cohort (*n* = 630), despite a similar unadjusted HR (1.83), the power decreased to 68.8% due to reduced sample size. Additionally, IPTW analysis preserved the full cohort size and yielded a power of 98.4%, further validating the robustness of our findings.

All analyses were performed using R software (version 4.3.2), with *p* < 0.05 indicating statistical significance.

## Results

**Figure 1.**
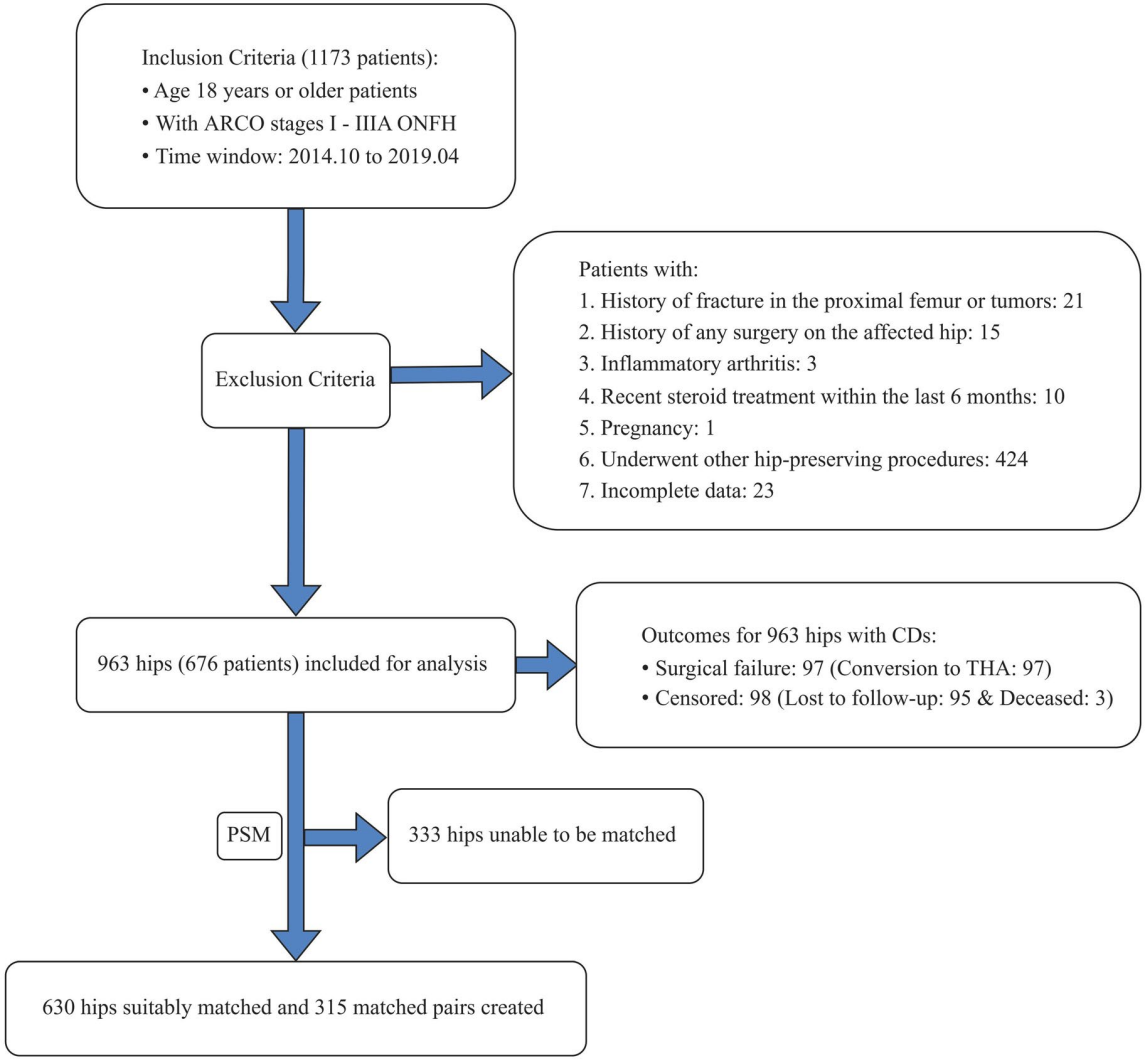
Flow chart for the selection of study subjects. ARCO, association Research Circulation Osseous; ONFH, osteonecrosis of the femoral head; PSM, propensity score matching.

As shown in Figure 1, a total of 1173 patients were identified for potential inclusion, and 497 were excluded according to the exclusion criteria. Finally, 963 eligible hips from 676 patients who underwent CD procedures were included, with 287 cases of simultaneous bilateral CD. Our cohort had a median age of 38.0 (31.0, 49.0) years, with a male predominance (83.0%). The majority of cases presented ARCO stage II (74.4%) ONFH. At baseline, the overall median SII was 586.81 [IQR 400.19, 1001.01], and there was a statistically significant difference in the median SII between the group of survival hips (567.74 [IQR 391.69, 940.84]) and the failure hips (914.90 [IQR 549.89, 1392.96], *p* < 0.001). The median follow-up time was 5.4 (4.0, 6.4) years. At the end of the follow-up, failure was observed in 97 cases of CD, and censoring was observed in 98 cases (95 lost to follow-up, 3 deaths). The cumulative incidence of failure was 10.6% (95% CI, 8.7%, 12.8%) at 5 years. We found a significant correlation between the SII index and the failure after CD (Mann–Whitney test: *p* < 0.001). When comparing the groups of survival hips and failure hips among baseline characteristics, differences reached or approached statistical significance regarding age, living place, aCCI, hyperlipidemia, ARCO stage, anesthesia method, ASA class and SII (all *p* < 0.1, Table 1), which were used for adjustment of RCS.

**Table 1.** Baseline characteristics of the study population*.

Variables	No. of overall hip with CD procedures *n* = 963	No. of^#^survival hip after CD procedures *n* = 866	No. of^#^failure hip after CD procedures *n* = 97	*p*
**Age (years)**	38.00 [31.00, 49.00]	37.00 [31.00, 48.00]	46.00 [34.00, 52.00]	**0.001**
< 47	693 (72.0%)	640 (74.9%)	53 (54.6%)	**<0.001**
≥ 47	270 (28.0%)	226 (26.1%)	44 (45.4%)	
**Gender**				0.396
Males	799 (83.0%)	722 (83.4%)	77 (79.4%)	
Females	164 (17.0%)	144 (16.6%)	20 (20.6%)	
**BMI (Kg/m2)**				0.944
< 18.5	17 (1.8%)	15 (1.7%)	2 (2.1%)	
18.5-23.9	335 (34.8%)	301 (34.8%)	34 (35.1%)	
24.0-27.9	418 (43.4%)	375 (43.3%)	43 (44.3%)	
≥ 28.0	193 (20.0%)	175 (20.2%)	18 (18.6%)	
**Living Place**				**0.083**
Rural	834 (86.6%)	756 (87.3%)	78 (80.4%)	
Urban	129 (13.4%)	110 (12.7%)	19 (19.6%)	
**Smoker**	162 (16.8%)	142 (16.4%)	20 (20.6%)	0.362
**Aetiology**				0.815
Steroid	509 (52.9%)	455 (52.5%)	54 (55.7%)	
Alcohol	273 (28.3%)	248 (28.6%)	25 (25.8%)	
Idiopathic	181 (18.8%)	163 (18.8%)	18 (18.6%)	
**aCCI**				**0.001**
0	669 (69.5%)	616 (71.1%)	53 (54.6%)	
1–2	256 (26.6%)	215 (24.8%)	41 (42.3%)	
≥ 3	38 (3.9%)	35 (4.0%)	3 (3.1%)	
**Preoperative comorbidities**				
Hyperlipidemia	316 (32.8%)	292 (33.7%)	24 (24.7%)	**0.095**
Hypertensive	104 (10.8%)	94 (10.9%)	10 (10.3%)	1.000
Diabetes	35 (3.6%)	32 (3.7%)	3 (3.1%)	0.988
Cerebrovascular disease	9 (0.9%)	9 (1.0%)	0 (0.0%)	0.651
Heart diseases	38 (3.9%)	35 (4.0%)	3 (3.1%)	0.857
Lung disease	10 (1.0%)	9 (1.0%)	1 (1.0%)	1.000
Liver disease	15 (1.6%)	13 (1.5%)	2 (2.1%)	1.000
Kidney disease	19 (2.0%)	16 (1.8%)	3 (3.1%)	0.652
Tumor	3 (0.3%)	3 (0.3%)	0 (0.0%)	1.000
Peripheral vascular disease	6 (0.6%)	6 (0.7%)	0 (0.0%)	0.887
Connective tissue disease	15 (1.6%)	13 (1.5%)	2 (2.1%)	1.000
**Total Hospital Stay (d)**	14.00 [11.00, 17.00]	14.00 [11.00, 17.00]	14.00 [10.00, 17.00]	0.391
**Surgical side**				0.154
Left	488 (50.7%)	446 (51.5%)	42 (43.3%)	
Right	475 (49.3%)	420 (48.5%)	55 (56.7%)	
**Thrombosis**	48 (5.0%)	44 (5.1%)	4 (4.1%)	0.869
**Anticoagulation** ^†^	869 (90.2%)	781 (90.2%)	88 (90.7%)	1.000
**With simultaneous contralateral THA**	193 (20.0%)	168 (19.4%)	25 (25.8%)	0.176
**Year of surgery**				0.822
2014	8 (0.8%)	2 (0.2%)	6 (6.2%)	
2015	289 (30.0%)	275 (31.8%)	14 (14.4%)	
2016	310 (32.2%)	265 (30.6%)	45 (46.4%)	
2017	165 (17.1%)	148 (17.1%)	17 (17.5%)	
2018	150 (15.6%)	140 (16.2%)	10 (10.3%)	
2019	41 (4.3%)	36 (4.2%)	5 (5.2%)	
**Surgeon experience (years)**				0.379
> 15	617 (64.1%)	559 (64.5%)	58 (59.8%)	
10-15	290 (30.1%)	255 (29.4%)	35 (36.1%)	
< 10	56 (5.8%)	52 (6.0%)	4 (4.1%)	
**ARCO stage**				**0.025**
I	163 (16.9%)	147 (17.0%)	16 (16.5%)	
II	716 (74.4%)	651 (75.2%)	65 (67.0%)	
IIIA	84 (8.7%)	68 (7.9%)	16 (16.5%)	
**Anesthesia method**				**0.021**
Regional	538 (55.9%)	495 (57.2%)	43 (44.3%)	
General	425 (44.1%)	371 (42.8%)	54 (55.7%)	
**ASA class**				**0.083**
I	8 (0.8%)	7 (0.8%)	1 (1.0%)	
II	894 (92.8%)	809 (93.4%)	85 (87.6%)	
≥ III	61 (6.3%)	50 (5.8%)	11 (11.3%)	
**Type of graft**				0.175
None	267 (27.7%)	237 (27.4%)	30 (30.9%)	
Autologous Bone Graft	186 (19.3%)	174 (20.1%)	12 (12.4%)	
Allogeneic Bone Graft	510 (53.0%)	455 (52.5%)	55 (56.7%)	
**SII Index**	586.81 [400.19, 1001.01]	567.74 [391.69, 940.84]	914.90 [549.89, 1392.96]	**<0.001**

* Values are median [Q1–Q3] for continuous variables and *n* (%) for categorical variables. Differences in characteristic were analyzed using Mann–Whitney tests for continuous variables and χ^2^-tests for categorical variables.

^#^ Survival was defined as CD without conversion to subsequent surgery at the last follow-up of patients.

^#^ Failure was defined as CD conversion to subsequent surgery at the last follow-up of patients.

^†^ The perioperative management of anticoagulation included systemic heparin and regional citrate; dose adjustment based on blood clotting tests.

CD, core decompression; BMI, body mass index; aCCI, age-adjusted Charlson’s comorbidity index; THA, total hip arthroplasty; ARCO, Association Research Circulation Osseous; ASA, American society of anesthesiologists; SII, systemic immune inflammation index.

**Figure 2.**
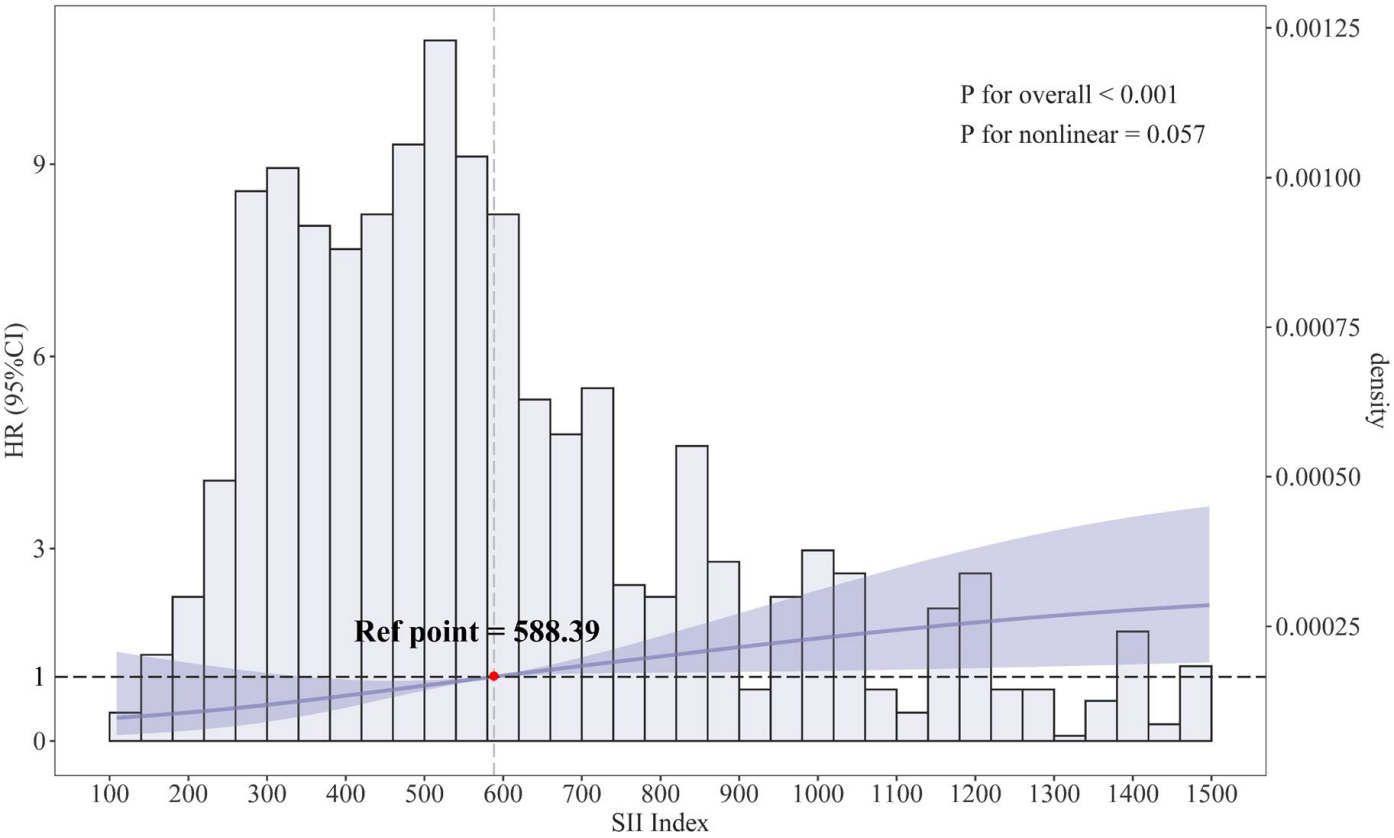
Dose-effect relationship between SII index and failure after core decompression (CD). Evaluated using a restricted cubic spline model with four knots and adjusted by all preoperative covariables with P-value < 0.1, including age, living place, age-adjusted charlson’s comorbidity index (aCCI), hyperlipidemia, association research circulation osseous (ARCO) stage, anesthesia method, American society of anesthesiologists (ASA) class, and SII index. P-nonlinearity showed no statistical significance, which was estimated using the likelihood ratio test comparing the restricted cubic spline model with the linear model. Relative risks were indicated by purple solid line and 95% CIs by purple shaded area, in which the reference point was 588.39 for the SII index. Blue shaded area showed the frequency distribution of SII index. SII, systemic immune inflammation index.

**Figure 3.**
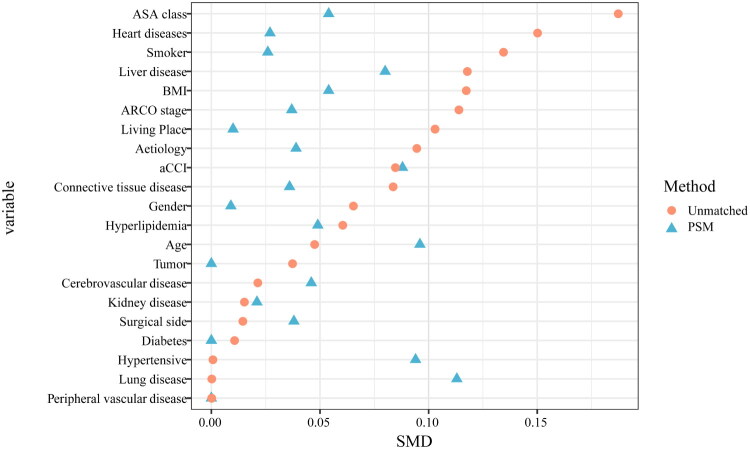
SMDs Distribution of preoperative covariables and their changes before and after PSM. SMD < 0.1 indicated adequate between-group balance. BMI, body mass index; aCCI, age-adjusted charlson’s comorbidity index; ARCO, association research circulation osseous; ASA, American society of anesthesiologists; PSM, propensity score match; SMD, standardized mean difference.

**Figure 4.**
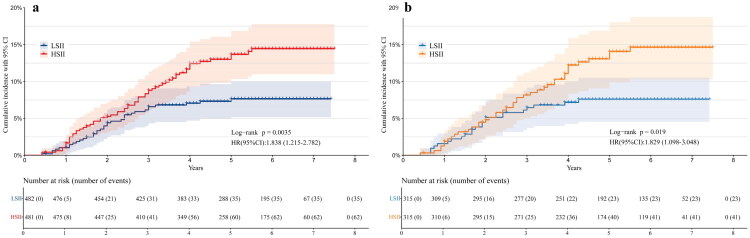
Cumulative incidence curves for patients undergoing CD. a. Before PSM, 963 hips were analyzed, comparing LSII (*n* = 482) with HSII (*n* = 481) for failure after CD. b. After PSM, 630 hips were analyzed, comparing LSII (*n* = 315) with HSII (*n* = 315). Confidence intervals (95%) are shaded. The number of patient hips at risk is shown at selected time points for each group. Cumulative incidence curves are evaluated using log-rank and Mantel-Cox analyzes. CD, core decompression; PSM, propensity score match; LSII, low systemic immune inflammation index; HSII, high systemic immune inflammation index; HR, hazard ratios.

**Figure 5.**
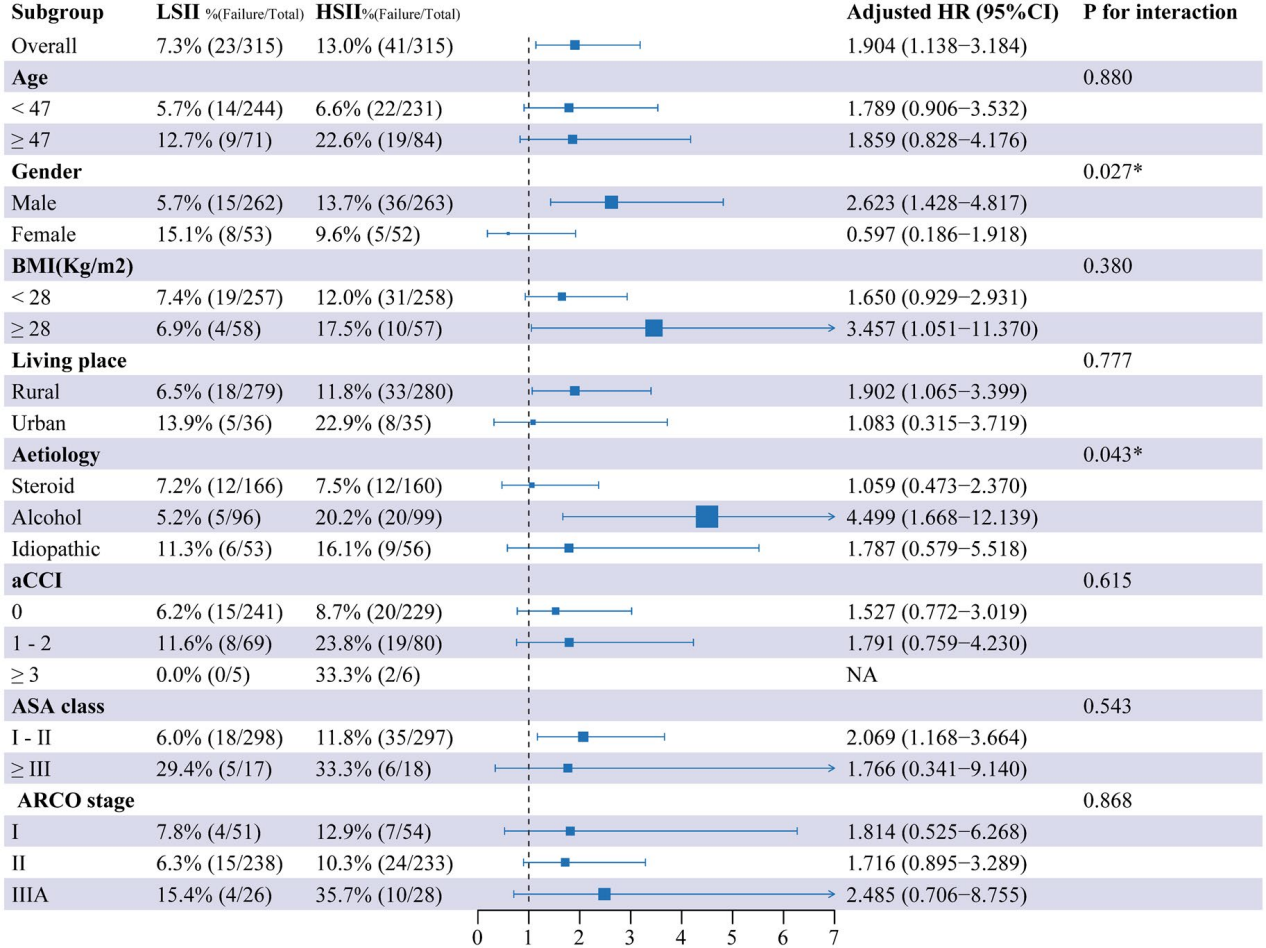
The association of HSII with the risk of failure after CD in various subgroups. HRs of failure after CD in relation to HSII were calculated using multivariate cox regression models, which were adjusted by surgeon, anesthesia method, type of graft, simultaneous contralateral THA, thrombosis, and anticoagulation. P for interaction was calculated using likelihood ratio tests. LSII, low systemic immune inflammation index; HSII, high systemic immune inflammation index; HR, hazard ratio; CI, confidence interval; BMI, body mass index; aCCI, age-adjusted charlson’s comorbidity index; ASA, American society of anesthesiologists; CI, confidence interval; CCI, charlson’s comorbidity index; ARCO, association research circulation osseous.

As shown in Figure 2, the adjusted RCS analysis indicates that the risk of failure after CD increases linearly with rising SII levels. The reference point, SII = 588.39, serves as the threshold at which the risk begins to rise significantly. A nonlinearity test yielded a *P*-value of 0.057, suggesting no statistically significant evidence of a nonlinear relationship. The cumulative incidence curves showed significant differences in failure rates at 5 years between the low SII and high SII groups, classified according to the reference point (13.7% vs. 7.6%) (Figure 4a). Before propensity score adjustment, the high SII group had a higher proportion of patients with current smoking, heart disease, and ASA class ≥ III (*p* < 0.05, Table 2), and the differences were well balanced after using PSM (Figure 3). After PSM, cumulative incidence curves between high SII group and low SII group still indicated significant differences in failure rate (14.1% vs. 7.6% at 5 years) (Figure 4b). After PSM and adjustment of perioperative factors, high SII was identified as independently associated with an increased risk of failure (HR, 1.90; 95% CI, 1.14, 3.18; *p* = 0.014), and general anesthesia (HR, 2.22; 95% CI, 1.28, 3.85; *p* = 0.005) would be significant risk factors for failure (Table 3). As shown in Figure 5, significant interactions and increased HRs of high SII associated with failure were observed in the male and alcoholic ONFH subgroups (*P* for interaction < 0.05).

**Table 2.** Comparison of the preoperative baseline characteristics of the subgroups of ‘low SII index’ and ‘high SII index’*.

Variables	Unmatched		SMD	*p*	After PSM		SMD	*p*
	LSII	HSII			LSII	HSII		
	482	481			315	315		
**Age (years)**			0.048	0.506			0.096	0.267
< 47	352 (73.0%)	341 (70.9%)			244 (77.5%)	231 (73.3%)		
≥ 47	130 (27.0%)	140 (29.1%)			71 (22.5%)	84 (26.7%)		
**Gender**			0.065	0.353			0.009	1.000
Males	394 (81.7%)	405 (84.2%)			262 (83.2%)	263 (83.5%)		
Females	88 (18.3%)	76 (15.8%)			53 (16.8%)	52 (16.5%)		
**BMI (Kg/m2)**			0.117	0.343			0.054	0.934
< 18.5	8 (1.7%)	9 (1.9%)			5 (1.6%)	5 (1.6%)		
18.5–23.9	168 (34.9%)	167 (34.7%)			113 (35.9%)	106 (33.7%)		
24.0–27.9	199 (41.3%)	219 (45.5%)			139 (44.1%)	147 (46.7%)		
≥ 28.0	107 (22.2%)	86 (17.9%)			58 (18.4%)	57 (18.1%)		
**Living Place**			0.103	0.133			0.010	1.000
Rural	409 (84.9%)	425 (88.4%)			279 (88.6%)	280 (88.9%)		
Urban	73 (15.1%)	56 (11.6%)			36 (11.4%)	35 (11.1%)		
**Smoker**	69 (14.3%)	93 (19.3%)	0.134	0.046	52 (16.5%)	49 (15.6%)	0.026	0.828
**Aetiology**			0.095	0.342			0.039	0.897
Steroid	259 (53.7%)	250 (52.0%)			166 (52.7%)	160 (50.8%)		
Alcohol	127 (26.3%)	146 (30.4%)			96 (30.5%)	99 (31.4%)		
Idiopathic	96 (19.9%)	85 (17.7%)			53 (16.8%)	56 (17.8%)		
**aCCI**			0.085	0.435			0.088	0.525
0	344 (71.4%)	325 (67.6%)			241 (76.5%)	229 (72.7%)		
1–2	121 (25.1%)	135 (28.1%)			69 (21.9%)	80 (25.4%)		
≥ 3	17 (3.5%)	21 (4.4%)			5 (1.6%)	6 (1.9%)		
**Preoperative comorbidities**								
Hyperlipidemia	165 (34.2%)	151 (31.4%)	0.061	0.384	97 (30.8%)	90 (28.6%)	0.049	0.601
Hypertensive	52 (10.8%)	52 (10.8%)	0.001	1.000	21 (6.7%)	29 (9.2%)	0.094	0.302
Diabetes	18 (3.7%)	17 (3.5%)	0.011	1.000	9 (2.9%)	9 (2.9%)	<0.001	1.000
Cerebrovascular disease	5 (1.0%)	4 (0.8%)	0.021	1.000	2 (0.6%)	1 (0.3%)	0.046	1.000
Heart diseases	12 (2.5%)	26 (5.4%)	0.150	0.031	5 (1.6%)	4 (1.3%)	0.027	1.000
Lung disease	5 (1.0%)	5 (1.0%)	<0.001	1.000	0 (0.0%)	2 (0.6%)	0.113	0.479
Liver disease	4 (0.8%)	11 (2.3%)	0.118	0.117	1 (0.3%)	3 (1.0%)	0.080	0.616
Kidney disease	9 (1.9%)	10 (2.1%)	0.015	0.996	7 (2.2%)	8 (2.5%)	0.021	1.000
Tumor	1 (0.2%)	2 (0.4%)	0.037	0.999	0 (0.0%)	0 (0.0%)	<0.001	NA
Peripheral vascular disease	3 (0.6%)	3 (0.6%)	<0.001	1.000	2 (0.6%)	2 (0.6%)	<0.001	1.000
Connective tissue disease	10 (2.1%)	5 (1.0%)	0.084	0.300	2 (0.6%)	3 (1.0%)	0.036	1.000
**Surgical side**			0.015	0.872			0.038	0.690
Left	246 (51.0%)	242 (50.3%)			158 (50.2%)	152 (48.3%)		
Right	236 (49.0%)	239 (49.7%)			157 (49.8%)	163 (51.7%)		
**ARCO Stage**			0.114	0.211			0.037	0.907
I	91 (18.9%)	72 (15.0%)			51 (16.2%)	54 (17.1%)		
II	353 (73.2%)	363 (75.5%)			238 (75.6%)	233 (74.0%)		
IIIA	38 (7.9%)	46 (9.6%)			26 (8.3%)	28 (8.9%)		
**ASA Class**			0.187	0.015			0.054	0.892
I	3 (0.6%)	5 (1.0%)			2 (0.6%)	1 (0.3%)		
II	459 (95.2%)	435 (90.4%)			298 (94.6%)	297 (94.3%)		
≥ III	20 (4.1%)	41 (8.5%)			15 (4.8%)	17 (5.4%)		

* Values are *n* (%) for categorical variables. PSM was applied for balancing the preoperative baseline characteristics between patients in the ‘LSII’ and ‘HSII’ groups, and SMD < 0.1 indicated adequate between-group balance.

PSM, propensity score match; SMD, standardized mean difference; SII, systemic immune inflammation index; BMI, body mass index; aCCI, age-adjusted Charlson’s comorbidity index; ARCO, Association Research Circulation Osseous; ASA, American society of anesthesiologists.

**Table 3.** Risk of failure after CD in relation to high SII index before and after propensity score matching*.

Variables	Before PSM		After PSM	
	HR (95% CI)	*p* value	HR (95% CI)	*p* value
**HSII**	1.88 (1.24–2.86)	**0.003**	1.90 (1.14 –3.18)	**0.014**
**Surgeon experience (years)**				
> 15	Ref		Ref	
10–15	1.43 (0.94–2.19)	0.098	1.21 (0.71–2.06)	0.481
< 10	0.98 (0.35–2.74)	0.971	0.81 (0.19–3.41)	0.770
**Anesthesia method**				
Regional	Ref		Ref	
General	1.70 (1.09–2.67)	**0.020**	2.22 (1.28–3.85)	**0.005**
**Type of graft**				
None	Ref		Ref	
Autologous bone graft	0.54 (0.27–1.08)	**0.083**	0.52 (0.21–1.31)	0.168
Allogeneic bone graft	0.96 (0.60–1.54)	0.865	1.22 (0.67–2.23)	0.521
**With simultaneous contralateral THA**	1.06 (0.62–1.82)	0.829	1.11 (0.58–2.13)	0.758
**Thrombosis**	0.66 (0.24–1.84)	0.432	0.25 (0.03–1.85)	0.175
**Anticoagulation**	1.01 (0.50–2.03)	0.977	0.84 (0.36–1.97)	0.695

* aHRs of failure after CD in relation to high SII index were estimated by using multivariable cox regression models adjusted by surgeon, anesthesia method, type of graft, simultaneous contralateral THA, thrombosis and anticoagulation.

CD, core decompression; HSII, high systemic immune inflammation index; THA, total hip arthroplasty; HR, hazard ratio; CI, confidence interval.

Overall, the results showed robustness in further IPTW and sensitivity analyses. In sensitivity analyses, by introducing robust variance estimation that accounted for pair matching after PSM and clustering among bilateral procedures in the model, the 95% confidence interval for hazard ratio (HR) estimate experienced a slight widening (95% CI, 1.14, 3.18 vs. 1.11, 3.26 vs. 1.10, 3.27). We also observed strong statistical significance and slightly varied HR estimate after incorporating lung disease into the model (1.90 vs. 1.92); stratifying by surgeon experience (1.90 vs.1.91), excluding participants who underwent simultaneous contralateral THA (1.90 vs. 2.64), with missing BMI (1.90 vs. 2.05), with CCI greater than or equal to 3 (1.90 vs. 1.81), and performing Landmark analysis (1.90 vs. 2.07) (Supplementary Table 1). After using IPTW, the differences in preoperative baseline characteristics were well balanced (Supplementary Table 2 and Supplementary Figure 3), and the HR estimate of high SII associated with failure was slightly downregulated (1.88 vs.1.78, Supplementary Table 3).

Among the 614 hips included in the post-hoc analysis, the mean mKA was 244.4° ± 42.7° (range: 158°–333°), with most classified as Grade 2–3 (200–299°, *n* = 434). JIC classification revealed that the majority of lesions extended over more than the medial two-thirds of the femoral head (type C1, *n* = 246; type C2, *n* = 327). Subgroup analysis showed no significant interaction between SII and mKA grade (interaction *p* = 0.228). A trend toward effect modification by location was observed, with a stronger association in JIC type C2 (HR = 2.31, 95% CI: 1.02, 5.22; *p* = 0.044) compared to C1 (HR = 1.76, 95% CI: 0.63, 4.95; *p* = 0.281; interaction *p* = 0.105; Supplementary Table 4). Location-stratified Cox models, adjusting for mKA using restricted cubic splines, confirmed that in type C2 hips (*n* = 306; events = 29), high SII remained independently associated with failure after CD (HR = 2.27; *p* = 0.043), whereas mKA was not significant (*p* > 0.7). In type C1 hips (*n* = 228; events = 18), no significant association with SII was detected, likely due to limited events and quasi-separation (Supplementary Table 5). In the BME-negative subgroup (*n* = 427), high SII remained significantly associated with CD failure (adjusted HR = 2.06; 95% CI: 1.12, 3.79; *p* = 0.021; Supplementary Table 1).

## Discussion

This prospective study focused on early to mid-stage ONFH patients, showing a positive dose-effect relationship between preoperative SII and failure after CD. After PSM, the 5-year failure rates significantly differed between high and low SII groups. High SII was independently associated with a 1.90-fold increased risk of failure after CD, with results remaining robust after IPTW and sensitivity analyses. Subgroup analyses revealed significant heterogeneities in gender and aetiology subgroups.

Our study revealed significant differences in preoperative SII levels between the survival group and the failure group. The median SII in the failure group was nearly 1.6 times higher than in the survival group (914.90 vs. 567.74), with an IQR (549.89–1392.96) highlighting considerable variability among individuals. In contrast, the survival group exhibited a lower median SII (567.74) and a narrower IQR (391.69–940.84), suggesting greater consistency in this cohort. Marked differences between groups at baseline suggest that SII may be linked to failure after CD, though further adjustments for confounding factors are needed to uncover the true relationship. To strengthen the reliability of our findings, we excluded patients with conditions known to elevate SII [17,29,30], such as infection, inflammatory bone disease, and cancer. We also conducted PSM using the RCS-derived threshold. Even after adjusting for baseline differences, the link between high SII and hip failure remained significant, indicating that this association holds independent of other potential confounders like age, gender, and etiology. While our main focus was on understanding factors tied to elevated SII, it’s worth noting that patients with lower SII levels generally had better outcomes. Factors potentially linked to lower SII include younger age, milder disease (e.g. earlier stages of ONFH), and an overall healthier baseline (e.g. lower BMI, no systemic inflammatory conditions, or lifestyle factors like non-smoking). Although these factors were partly addressed through PSM, their combined role in reducing systemic inflammation cannot be fully discounted. This finding aligns with mounting evidence that systemic inflammation hinders bone repair and drives osteonecrosis progression. Elevated SII, which reflects a pro-inflammatory state driven by neutrophils and platelets, may worsen microvascular dysfunction and subchondral bone resorption, ultimately reducing the effectiveness of CD [31].

Assessment of prognostic outcomes based on patient peripheral blood parameters remains an underexplored aspect of ONFH management. In this study, we explored the utility of the SII in prognostic outcomes for patients undergoing CD, aiming to enrich current understanding in this domain. Several strengths of this predictor need to be elucidated. Firstly, relevant parameters can be easily retrieved from patient medical records, eliminating the need for direct physical evaluations, a feature particularly beneficial in referral centers. Secondly, the SII, as an independent standard laboratory measure that is not dependent on imaging, could serve as a more objective tool to guide surgical decisions, notably in cases where the choice between hip preservation and replacement is contentious [32]. Thirdly, by integrating routine blood test results and baseline patient characteristics [2], surgeons can identify individuals at an early stage who are at risk of ONFH progression yet remain asymptomatic. Further recommendations for imaging and appropriate presurgical management can be abundant for secondary prevention of ONFH, while the predictive precision of SII warrants further validation in a wider ONFH population.

The SII is considered to be a comprehensive indicator for predicting the risk of inflammation, and the persistent chronic inflammatory response in necrotic areas may contribute predominantly to failure after CD [18,31]. As the disruption of the balance between pro-inflammatory activation and anti-inflammatory mechanisms, innate (e.g. neutrophils) and adaptive (e.g. T cells and B cells) immune cells are continuously recruited to necrotic areas, where they release more inflammatory mediators, creating a feedback loop that exacerbates inflammation [33]. This increased inflammatory state and accumulation of inflammatory cells can lead to increased bone resorption, decreased bone formation, and impaired microvascular blood flow [31,34]. Furthermore, aberrant platelet aggregation of inflammatory origin can cause localized microvascular ischemia, hypoxia, and microthrombosis, affecting the blood supply to the necrotic area [35].

Significant heterogeneity and elevated HR of high SII associated with failure after CD were observed in subgroups of male and alcoholic ONFH. These findings indicate that significant interactions between high SII and certain specific conditions that have been widely discussed [28,36] contributed to driving the effects estimates. Patients with alcoholic ONFH have the potential to achieve the greatest beneficial effects through abstinence, yet for immutable gender conditions, high-risk individuals may also benefit more from secondary prevention strategies such as early screening and intervention to delay CD failure or even avoid surgery altogether.

To assess the robustness of our findings to potential unmeasured confounding, we calculated the E-value [37,38]. Given the observed HR of 1.90 (95% CI: 1.14, 3.18), the E-value for the point estimate was 3.21, and for the lower bound of the confidence interval was 1.54. This indicates that an unmeasured confounder would need to be associated with both the exposure (SII) and the outcome (CD failure) by a risk ratio of at least 3.21, beyond the measured covariates, to fully account for the observed association. Additionally, although the effect size of SII on conversion risk remained consistent across analytical methods, the statistical power varied depending on the analytical approach. Specifically, propensity score matching, while enhancing covariate balance, led to a reduction in sample size and power (68.8%), potentially increasing the risk of type II error. In contrast, IPTW analysis preserved the entire cohort and yielded sufficient power (98.4%), reinforcing the reliability of our findings. These results highlight the inherent trade-off between confounding control and statistical efficiency when choosing between matching and weighting approaches.

Strengths of our study include its prospective design, a relatively large sample size, control of possible preoperative confounders, and interaction analyzes to address potential population heterogeneity. The hazard ratio estimates were overall robust under rigorous sensitivity analyses. Nevertheless, several limitations warrant mention.

First, our outcome, time to surgical failure, defined as conversion of CD to subsequent surgery, might not fully reflect the survival of CD. The decision to undergo subsequent surgery can be influenced by a variety of factors, such as economic status, pain tolerance, and functional demand, which were not evaluated in this study. However, employing subsequent surgery as an objective outcome measure mitigates the inherent biases inherent in subjective assessments. For instance, subjective assessment of the degree of collapse based on MRI cannot precisely determine the timing of collapse and is subject to unavoidable inter-observer variability. Moreover, several studies have adopted progression to subsequent surgery as an objective evaluation criterion without diminishing its clinical value [24,39]. Future studies should aim to integrate objective and subjective outcomes for a more comprehensive evaluation. Second, while we combined visual inspection, the Maximum Selected Rank Statistics Method, and Threshold Effect Analysis to accurately identify inflection points in the RCS curves, inherent variations—such as clinical/epidemiological factors (e.g. race, demographics, prevalence) and postoperative strategies (e.g. functional exercise, weight-bearing timing, concurrent medications) [40,41]—may limit the cut-off value’s generalizability. Future studies should adjust for these confounders to derive a more broadly applicable threshold. Third, this study was conducted at a single center and involved the recruitment of patients from a tertiary referral medical center for orthopedic. Therefore, it may have introduced a selection bias toward patients with complex conditions or those referred by experienced primary care physicians, being less representative of all patients eligible for CD procedures. Fourth, while Landmark analysis strengthened the temporal validity of our findings, residual confounding from unmeasured factors (e.g. microvascular necrosis extent before CD [32]) may still exist. Future studies with serial biomarker measurements are needed to fully exclude reverse causality. Fifth, although the post-hoc analyses demonstrate a predictive effect of high SII in pre-collapse hips (particularly in JIC C2 after adjusting for lesion size), the conclusions are constrained by the number of events and by the surgical nature of the cohort. Further validation in lesion-specific cohorts and in asymptomatic cohorts is warranted. Finally, due to the limited number of outcome events and lack of external validation, this study did not attempt to build a multivariable prognostic model such as a nomogram, which would require a substantially larger event-per-variable ratio to ensure predictive accuracy and generalizability.

## Conclusion

The present study found for the first time that preoperative high SII was independently associated with a 90% higher risk of failure after CD for early to mid-stage ONFH. Additionally, increased risk estimates were observed in male and alcoholic ONFH subgroups, indicating a need for earlier screening and intervention in these populations. These findings could be considered when evaluating surgical indications and providing preoperative counseling to patients.

## Supplementary Material

Supplemental Material

Supplementary.docx

## Data Availability

The datasets used and/or analyzed during the current study are available from the corresponding author on reasonable request.
