# Supplementary figures and images for: Association of the systemic immune inflammation index with failure after core decompression for osteonecrosis of the femoral head: a prospective time-to-event analysis
Source: Ann Med. 2025 Oct 6;57(1):2566867. doi: 10.1080/07853890.2025.2566867 (PMC12502117; doi:10.1080/07853890.2025.2566867)

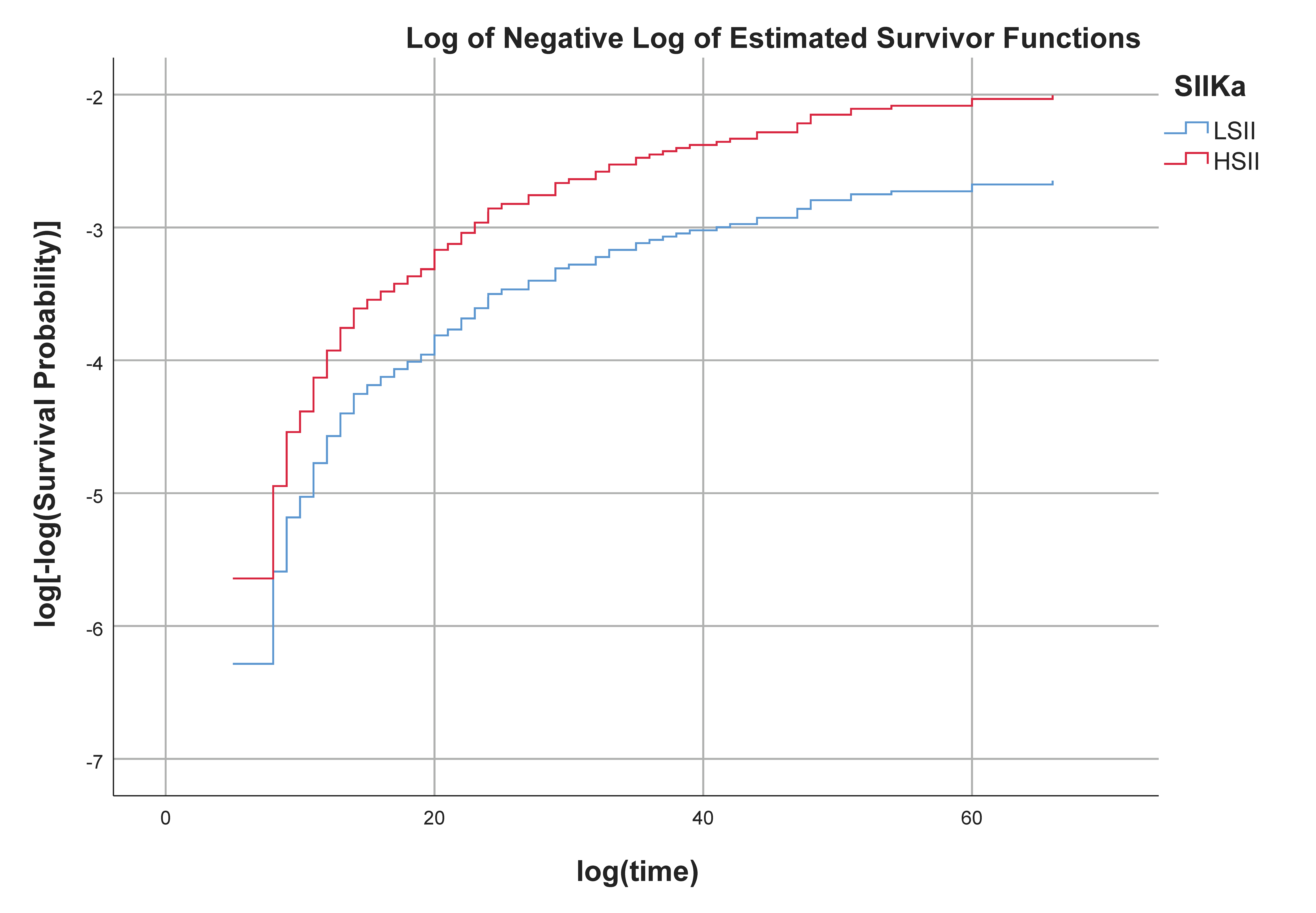

Supplement: Supplemental Material [file IANN_A_2566867_SM9098.zip › Supplementary Figures/Supplementary Figure 1.tif]

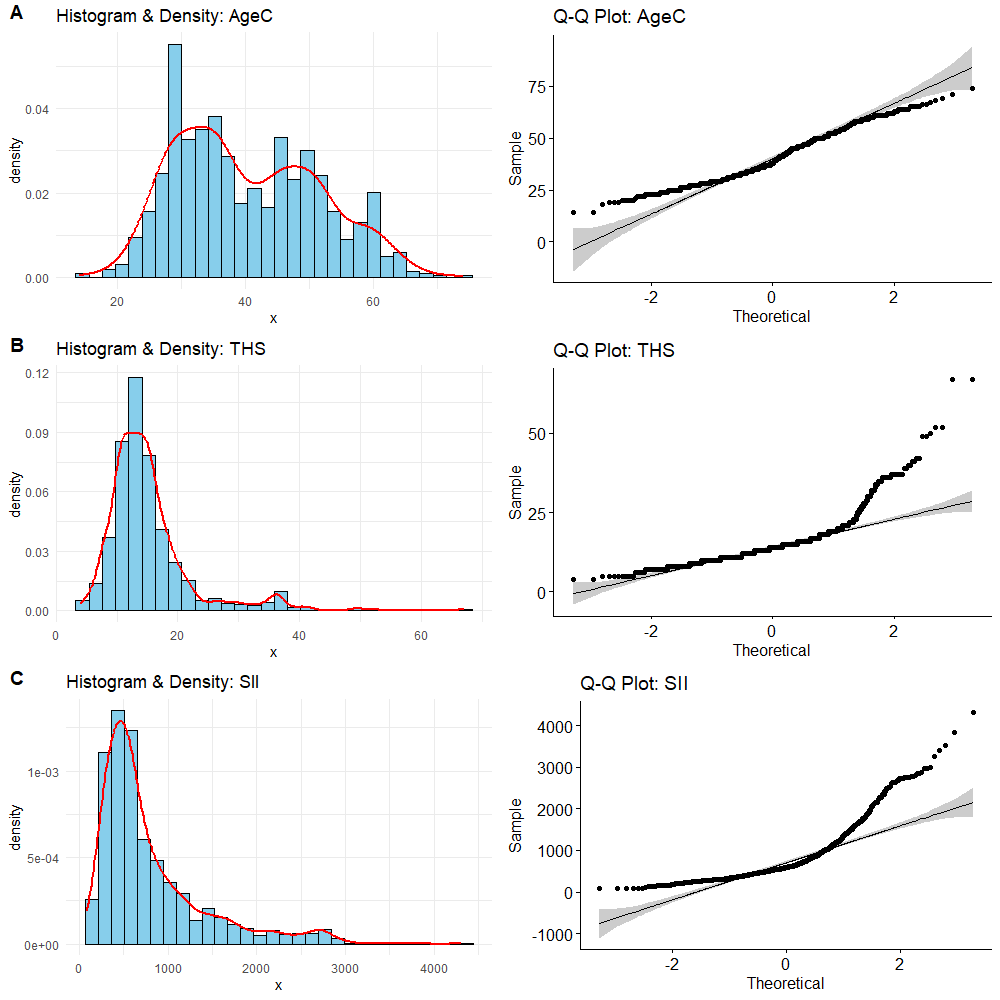

Supplement: Supplemental Material [file IANN_A_2566867_SM9098.zip › Supplementary Figures/Supplementary Figure 4.tiff]
