# Supplementary material for: Association of the systemic immune inflammation index with failure after core decompression for osteonecrosis of the femoral head: a prospective time-to-event analysis
Source: Ann Med. 2025 Oct 6;57(1):2566867. doi: 10.1080/07853890.2025.2566867 (PMC12502117; doi:10.1080/07853890.2025.2566867)
Supplement: Supplementary.docx [file IANN_A_2566867_SM3646.docx]

**Supplementary Table 1. Sensitivity analyses and post-hoc analysis for testing the risk of failure after CD in relation to HSII on the post-PSM data.**

| **Group** | **LSII** |  | **HSII** |  | **^#^Unadjusted** | |  | **^#^Adjusted** | |  |
| --- | --- | --- | --- | --- | --- | --- | --- | --- | --- | --- |
|  | **Failure / Total** |  | **Failure / Total** |  | **HR (95% CI)** | **P** |  | **HR (95% CI)** | **P** |  |
| Original cohort after PSM | 7.3% (23/315) |  | 13.0% (41/315) |  | 1.83 (1.09-3.05) | 0.021 |  | 1.90 (1.14-3.18) | 0.014 |  |
| Adjust for pair matching^§^ | 7.3% (23/315) |  | 13.0% (41/315) |  | 1.83 (1.09-3.08) | 0.023 |  | 1.90 (1.11-3.26) | 0.019 |  |
| Adjust for bilateral CD* | | 7.3% (23/315) |  | 13.0% (41/315) |  | 1.83 (1.07-3.12) | 0.026 |  | 1.90 (1.10-3.27) | 0.020 |
| Incorporate lung disease | | 7.3% (23/315) |  | 13.0% (41/315) |  | 1.83 (1.09-3.05) | 0.021 |  | 1.92 (1.12-3.30) | 0.019 |
| Stratify by surgeon experience | 7.3% (23/315) |  | 13.0% (41/315) |  | 1.82 (1.09-3.03) | 0.022 |  | 1.91 (1.14-3.20) | 0.014 |  |
| Patients without simultaneous contralateral THA | 5.4% (14/259) |  | 13.2% (34/257) |  | 2.54 (1.36-4.74) | 0.003 |  | 2.64 (1.41-4.94) | 0.002 |  |
| Patients without missing BMI | 6.6% (20/302) |  | 12.7% (38/300) |  | 1.97 (1.14-3.38) | 0.014 |  | 2.05 (1.19-3.55) | 0.009 |  |
| Patients without aCCI ≥ 3 | 7.4% (23/310) |  | 12.6% (39/309) |  | 1.74 (1.04-2.91) | 0.035 |  | 1.81 (1.08-3.04) | 0.025 |  |
| Landmark analysis^†^ | 5.8% (18/309) |  | 11.4% (35/307) |  | 2.01 (1.14-3.55) | 0.016 |  | 2.07 (1.17-3.67) | 0.013 |  |
| BME-negative analysis^I^ | 8.4% (19/225) |  | 15.8% (32/202) |  | 1.91 (1.07-3.42) | 0.029 |  | 2.06 (1.12-3.79) | 0.021 |  |
| ^#^Unadjusted: using univariate cox regression analysis for the post-PSM data. ^#^Adjusted: using multivariate cox regression analysis for the post-PSM data with adjustment for perioperative factors. ^§^HRs were adjusted by incorporating a robust variance estimation that accounted for pair matching after PSM in the model. *HRs were adjusted at the patient level for clustering among bilateral procedures by incorporating a robust variance estimation in the model. ^†^Landmark analysis performed at 12 months post-operation, excluding early failures (n=14). ^I^BME-negative analysis was performed by excluding hips with bone marrow edema (n=187) on preoperative MRI in the post-PSM cohort. CD, core decompression; HSII, high systemic immune inflammation index; LSII low systemic immune inflammation index; PSM, propensity score match; HR, hazard ratio; CI, confidence interval; THA, total hip arthroplasty; BMI, body mass index; aCCI, age-adjusted Charlson's comorbidity index; BME, bone marrow edema. | | | | | | | | | |  |

| **Supplementary Table 2. Comparison of the preoperative baseline characteristics of the groups of ‘LSII’ and ‘HSII’^1^** | | | | | | | | |
| --- | --- | --- | --- | --- | --- | --- | --- | --- |
| **Variables** | **Unmatched** | | **SMD** | **p** | **After IPTW** | | **SMD** | **p** |
|  | **LSII** | **HSII** |  |  | **LSII** | **HSII** |  |  |
|  | **482** | **481** |  |  | **966.3** | **960.3** |  |  |
| **Age (years)** |  |  | 0.048 | 0.506 |  |  | 0.003 | 0.960 |
| ≤47 | 352 (73.0%) | 341 (70.9%) |  |  | 703.9 (72.8%) | 698.1 (72.7%) |  |  |
| >47 | 130 (27.0%) | 140 (29.1%) |  |  | 262.4 (27.2%) | 262.2 (27.3%) |  |  |
| **Gender** |  |  | 0.065 | 0.353 |  |  | 0.001 | 0.992 |
| Males | 394 (81.7%) | 405 (84.2%) |  |  | 804.0 (83.2%) | 798.8 (83.2%) |  |  |
| Females | 88 (18.3%) | 76 (15.8%) |  |  | 162.3 (16.8%) | 161.5 (16.8%) |  |  |
| **BMI (Kg/m2)** |  |  | 0.117 | 0.343 |  |  | 0.013 | 0.998 |
| < 18.5 | 8 (1.7%) | 9 (1.9%) |  |  | 15.5 (1.6%) | 16.3 (1.7%) |  |  |
| 18.5-23.9 | 168 (34.9%) | 167 (34.7%) |  |  | 335.4 (34.7%) | 335.0 (34.9%) |  |  |
| 24.0-27.9 | 199 (41.3%) | 219 (45.5%) |  |  | 425.1 (44.0%) | 417.4 (43.5%) |  |  |
| ≥ 28.0 | 107 (22.2%) | 86 (17.9%) |  |  | 190.3 (19.7%) | 191.6 (19.9%) |  |  |
| **Living Place** |  |  | 0.103 | 0.133 |  |  | 0.004 | 0.952 |
| Rural | 409 (84.9%) | 425 (88.4%) |  |  | 835.7 (86.5%) | 831.9 (86.6%) |  |  |
| Urban | 73 (15.1%) | 56 (11.6%) |  |  | 130.6 (13.5%) | 128.4 (13.4%) |  |  |
| **Smoker** | 69 (14.3%) | 93 (19.3%) | 0.134 | 0.046 | 165.6 (17.1%) | 162.9 (17.0%) | 0.005 | 0.946 |
| **Aetiology** |  |  | 0.095 | 0.342 |  |  | 0.006 | 0.995 |
| Steroid | 259 (53.7%) | 250 (52.0%) |  |  | 508.3 (52.6%) | 507.1 (52.8%) |  |  |
| Alcohol | 127 (26.3%) | 146 (30.4%) |  |  | 278.3 (28.8%) | 277.0 (28.8%) |  |  |
| Idiopathic | 96 (19.9%) | 85 (17.7%) |  |  | 179.8 (18.6%) | 176.3 (18.4%) |  |  |
| **aCCI** |  |  | 0.085 | 0.435 |  |  | 0.010 | 0.990 |
| 0 | 344 (71.4%) | 325 (67.6%) |  |  | 673.6 (69.7%) | 673.0 (70.1%) |  |  |
| 1-2 | 121 (25.1%) | 135 (28.1%) |  |  | 256.9 (26.6%) | 251.3 (26.2%) |  |  |
| ≥3 | 17 (3.5%) | 21 (4.4%) |  |  | 35.8 (3.7%) | 36.0 (3.8%) |  |  |
| **Preoperative comorbidities** | |  |  |  |  |  |  |  |
| Hyperlipidemia | 165 (34.2%) | 151 (31.4%) | 0.061 | 0.384 | 312.2 (32.3%) | 312.7 (32.6%) | 0.005 | 0.934 |
| Hypertensive | 52 (10.8%) | 52 (10.8%) | 0.001 | 1 | 99.4 (10.3%) | 99.7 (10.4%) | 0.003 | 0.960 |
| Diabetes | 18 (3.7%) | 17 (3.5%) | 0.011 | 1 | 33.7 (3.5%) | 33.8 (3.5%) | 0.002 | 0.977 |
| Cerebrovascular disease | 5 (1.0%) | 4 (0.8%) | 0.021 | 1 | 8.4 (0.9%) | 8.2 (0.9%) | 0.001 | 0.983 |
| Heart diseases | 12 (2.5%) | 26 (5.4%) | 0.150 | 0.031 | 39.3 (4.1%) | 38.5 (4.0%) | 0.003 | 0.971 |
| Lung disease | 5 (1.0%) | 5 (1.0%) | <0.001 | 1 | 8.8 (0.9%) | 9.2 (1.0%) | 0.005 | 0.933 |
| Liver disease | 4 (0.8%) | 11 (2.3%) | 0.118 | 0.117 | 17.0 (1.8%) | 15.3 (1.6%) | 0.013 | 0.870 |
| Kidney disease | 9 (1.9%) | 10 (2.1%) | 0.015 | 0.996 | 19.0 (2.0%) | 19.1 (2.0%) | 0.002 | 0.978 |
| Tumor | 1 (0.2%) | 2 (0.4%) | 0.037 | 0.999 | 2.7 (0.3%) | 3.0 (0.3%) | 0.005 | 0.945 |
| Peripheral vascular disease | 3 (0.6%) | 3 (0.6%) | <0.001 | 1 | 5.7 (0.6%) | 6.0 (0.6%) | 0.003 | 0.957 |
| Connective tissue disease | 10 (2.1%) | 5 (1.0%) | 0.084 | 0.3 | 14.5 (1.5%) | 13.4 (1.4%) | 0.009 | 0.897 |
| **Surgical side** |  |  | 0.015 | 0.872 |  |  | 0.003 | 0.967 |
| Left | 246 (51.0%) | 242 (50.3%) |  |  | 491.5 (50.9%) | 489.8 (51.0%) |  |  |
| Right | 236 (49.0%) | 239 (49.7%) |  |  | 474.8 (49.1%) | 470.5 (49.0%) |  |  |
| **ARCO Stage** |  |  | 0.114 | 0.211 |  |  | 0.005 | 0.998 |
| I | 91 (18.9%) | 72 (15.0%) |  |  | 160.5 (16.6%) | 158.7 (16.5%) |  |  |
| II | 353 (73.2%) | 363 (75.5%) |  |  | 722.3 (74.7%) | 717.5 (74.7%) |  |  |
| III | 38 (7.9%) | 46 (9.6%) |  |  | 83.5 (8.6%) | 84.1 (8.8%) |  |  |
| **ASA Class** |  |  | 0.187 | 0.015 |  |  | 0.005 | 0.997 |
| I | 3 (0.6%) | 5 (1.0%) |  |  | 8.2 (0.9%) | 8.1 (0.8%) |  |  |
| II | 459 (95.2%) | 435 (90.4%) |  |  | 898.5 (93.0%) | 891.8 (92.9%) |  |  |
| ≥ III | 20 (4.1%) | 41 (8.5%) |  |  | 59.6 (6.2%) | 60.4 (6.3%) |  |  |
| ^1^Values are n (%) for categorical variables. IPTW was applied for balancing the preoperative baseline characteristics between patients in the “LSII” and “HSII” groups, and SMD < 0.1 indicated adequate between-group balance. | | | | | | | | |
| LSII, low systemic immune inflammation index; HSII, high systemic immune inflammation index; IPTW, inverse probability treatment weighting; SMD, standardized mean difference; BMI, body mass index; aCCI, age-adjusted Charlson's comorbidity index; ARCO, Association Research Circulation Osseous; ASA, American society of anesthesiologists. | | | | | | | | |

| **Supplementary Table 3.** **Risk of failure after CD in relation to HSII before and after IPTW*** | | | | | | | | | | | |  |
| --- | --- | --- | --- | --- | --- | --- | --- | --- | --- | --- | --- | --- |
| **Variables** | | | **Unmatched** | | | | | **After IPTW** | | | |  |
|  |  |  | **HR (95% CI)** | | | **P value** | | **HR (95% CI)** | | **P value** | |  |
|  |  |  |  |  |  |  |  |  |  |  |  |  |
| **HSII** | | | 1.88 (1.24 - 2.86) | | | **0.003** | | 1.78 (1.15 -2.76) | | **0.009** | |  |
| **Surgeon experience (years)** | | |  | | |  | |  | |  | |  |
| >15 | | | Ref | | |  | | Ref | |  | |  |
| 10-15 | | | 1.43 (0.94 - 2.19) | | | 0.098 | | 1.42 (0.93 - 2.18) | | 0.108 | |  |
| <10 | | | 0.98 (0.35 - 2.74) | | | 0.971 | | 1.29 (0.48 - 3.51) | | 0.609 | |  |
| **Anesthesia method** | | |  | | |  | |  | |  | |  |
| Regional | | | Ref | | |  | | Ref | |  | |  |
| General | | | 1.70 (1.09 - 2.67) | | | **0.020** | | 1.62 (1.01 - 2.62) | | **0.047** | |  |
| **Type of graft** | | |  | | |  | |  | |  | |  |
| None | | | Ref | | |  | | Ref | |  | |  |
| Autologous Bone Graft | | | 0.54 (0.27 - 1.08) | | | **0.083** | | 0.52 (0.26 - 1.06) | | 0.074 | |  |
| Allogeneic Bone Graft | | | 0.96 (0.60 - 1.54) | | | 0.865 | | 0.99 (0.62 – 1.60) | | 0.991 | |  |
| **With simultaneous contralateral THA** | | | 1.06 (0.62 - 1.82) | | | 0.829 | | 1.14 (0.65 - 2.01) | | 0.638 | |  |
| **Thrombosis** | | | 0.66 (0.24 - 1.84) | | | 0.432 | | 0.59 (0.22 - 1.62) | | 0.307 | |  |
| **Anticoagulation** | | | 1.01 (0.50 - 2.03) | | | 0.977 | | 1.01 (0.52 - 1.97) | | 0.973 | |  |
| *HRs of failure after CD in relation to HSII index were estimated by using multivariable cox regression models adjusted by surgeon experience, anesthesia method, type of graft, simultaneous contralateral THA, thrombosis and anticoagulation. | | | | | | | | | | | |  |
| CD, core decompression; HSII, high systemic immune inflammation index; IPTW, inverse probability treatment weighting; HR, hazard ratio; CI, confidence interval; THA, total hip arthroplasty. | | | | | | | | | | | |  |
| **Supplementary Table 4. Subgroup analyses by mKA grade and JIC type with adjustment for perioperative covariates.** | | | | | | | | | | |  |  |
| **Variable** | **Level** | **Count** | | **Events** | **HR (95% CI)** | | **P value** | | **P for interaction** | |  |  |
| Overall |  | 561 | | 49 |  | |  | |  | |  |  |
| mKA Grade | Grade 1 (<200°) | 119 | | 7 | 4.31 (0.50–36.95) | | 0.183 | | 0.228 | |  |  |
| mKA Grade | Grade 2 (200–249°) | 171 | | 20 | 0.96 (0.37–2.49) | | 0.941 | |  | |  |  |
| mKA Grade | Grade 3 (250–299°) | 222 | | 9 | 2.47 (0.62–9.92) | | 0.201 | |  | |  |  |
| mKA Grade | Grade 4 (≥300°) | 49 | | 13 | 6.16 (0.94–40.40) | | 0.058 | |  | |  |  |
| JIC | A | 9 | | 0 | — | | No events in stratum | | 0.105 | |  |  |
| JIC | B | 18 | | 2 | — | | Not estimable | |  | |  |  |
| JIC | C1 | 228 | | 18 | 1.76 (0.63–4.95) | | 0.281 | |  | |  |  |
| JIC | C2 | 306 | | 29 | 2.31 (1.02–5.22) | | 0.044 | |  | |  |  |
| HR, hazard ratio; CI, confidence interval; mKA, modified Kerboul angle; JIC, Japanese Investigation Committee. | | | | | | | | | | |  |  |

| **Supplementary Table 5. Location-stratified Cox models, adjusting mKA continuously via restricted cubic splines.** | | | | | | | | |
| --- | --- | --- | --- | --- | --- | --- | --- | --- |
| **JIC type** | **N** | **Events** | **SII coefficient** | **SII HR** | **SII 95% CI** | **SII P value** | **mKA overall P** | **mKA nonlinearity P** |
| C1 | 228 | 18 | 0.5239 | 1.69 | 1.69 (0.64–4.47) | 0.2918 | 0.6517 | 0.9139 |
| C2 | 306 | 29 | 0.8178 | 2.27 | 2.27 (1.02–5.01) | 0.0433 | 0.7401 | 0.9272 |
| Notes: Limited events; quasi-separation for a rare covariate level; Same rcs(mKA,3) in both strata; perioperative covariates adjusted. SII, systemic immune inflammation index; HR, hazard ratio; CI, confidence interval; mKA, modified Kerboul angle; JIC, Japanese Investigation Committee. | | | | | | | | |
|  |  |  |  |  |  |  |  |  |

**Supplementary** **Figure 1** Log-log plots supported the proportional hazard assumption in multivariate Cox proportional hazards model. The graph of the log(-log(primary outcome)) versus log of survival time resulted in parallel lines.

**Supplementary** **Figure 2** Schoenfeld residual plots supported the proportional hazard assumption in multivariate Cox proportional hazards model. The graph of the Schoenfeld residual plots confirmed that HRs were constant from time since CD through the entire observable follow-up period.

**Supplementary** **Figure 3** SMDs distribution of preoperative covariables and their changes before and after IPTW. SMD < 0.1 indicated adequate between-group balance. SMD, standardized mean difference; IPTW, inverse probability treatment weighting; BMI, body mass index; aCCI, age-adjusted Charlson's comorbidity index; ARCO, Association Research Circulation Osseous; ASA, American society of anesthesiologists.

**Supplementary** **Figure 4** Normality assessment for continuous variables. Each variable was evaluated for normality using histogram with kernel density overlay (left) and Q–Q plot (right). (A) Age shows an approximately symmetric distribution, but formal tests indicated deviation from normality. (B) Total hospital stay exhibits right skewness with evident departure from the normal distribution in both visual and statistical assessments. (C) SII is heavily right-skewed, clearly deviating from normality. All variables failed Shapiro–Wilk and Kolmogorov–Smirnov tests (P < 0.001), and were conservatively treated as non-normally distributed.
